# Supplementary material for: Mosquito vector ecologies are destabilizing as a result of climate change
Source: bioRxiv. 2025 Sep 17:2025.09.15.676177. Preprint. [Version 1] doi: 10.1101/2025.09.15.676177 (PMC12458471; doi:10.1101/2025.09.15.676177)
Supplement: Supplement 1 — S1: Average maps for all half-decade averages, all three species S2: Difference maps for all half-decade averages (to 1980-4), all three species S3: Difference maps for all half-decade averages (consecutive) S4: Seasonality and anomaly heatmaps, all three species S5: 3D Population overlaid with FNR difference from 2010 to 2020 [file media-1.pdf]

Mosquito vector ecologies are destabilizing as a result of climate change

Supplementary Figures

Authors

Evan J. Curcio (1,2,4), Kai Xu (2,3), Harutyun Sahakyan (2), Yuri I. Wolf (2), Elizabeth A. Kelvin (1,4,5), Nash D. Rochman* (1,2,4)

Affiliations

1 - City University of New York Graduate School of Public Health and Health Policy, Department of Epidemiology and Biostatistics, New York, NY, United States

2 - Computational Biology Branch, Division of Intramural Research, National Library of Medicine, National Institutes of Health, Bethesda, MD, United States

3 - Courant Institute of Mathematical Sciences, New York University, New York, NY, United States

4 - Institute for Implementation Science in Population Health, City University of New York School of Public Health, New York, NY

5 - Department of Occupational Health, Epidemiology & Prevention, Donald and Barbara Zucker School of Medicine at Hofstra University/Northwell Health, Hempstead, NY, USA

* Corresponding author: nash.rochman@sph.cuny.edu

Figure S1:

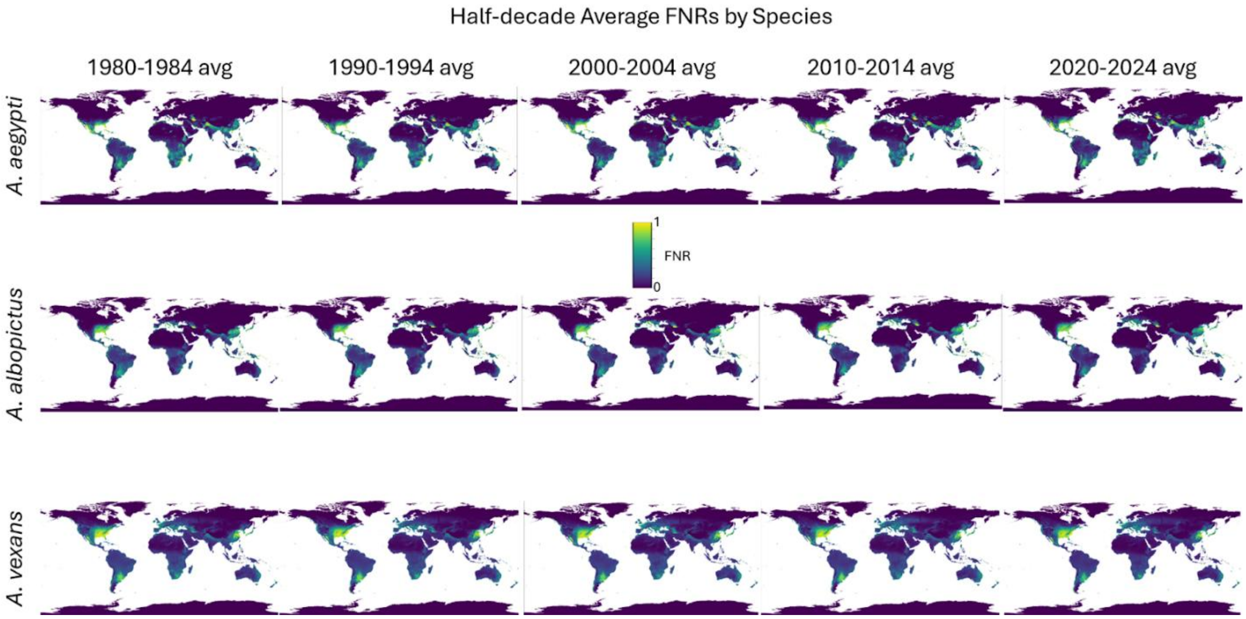

Supplementary Figure 1: Half-decade average false negative rates (FNRs) by species. Daily FNR values from each half-decade were averaged for each of the five timespans shown.

Figure S2:

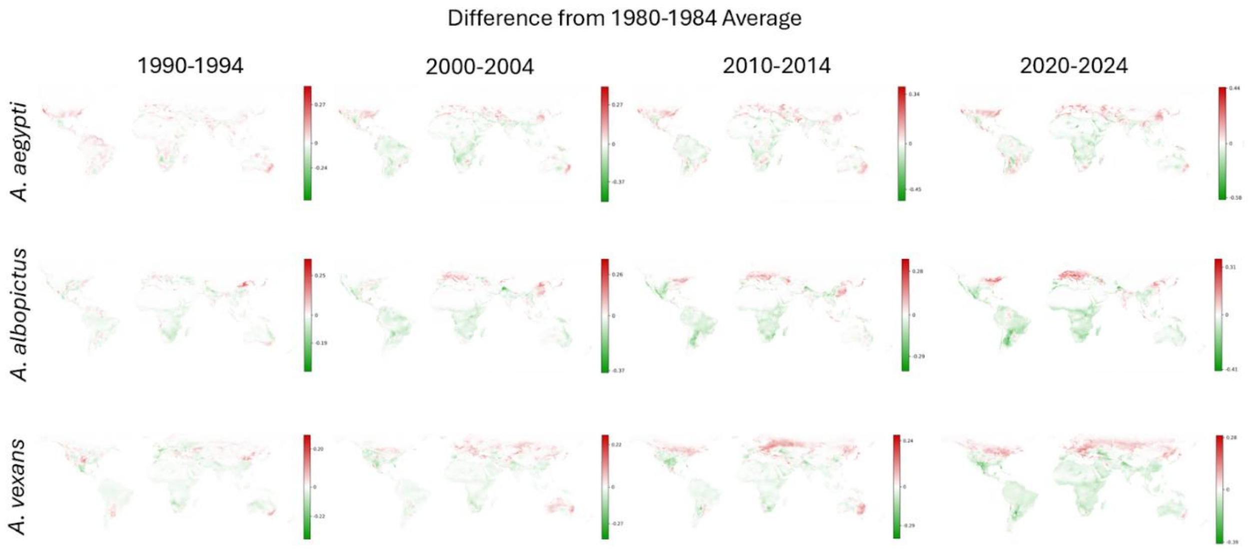

Supplementary Figure 2: Difference maps from 1980-1984 average map. Differences were taken between the 1980-1984 average value and all later timespans. Red indicates an increase in mosquito risk at a given location, while green indicates a decrease.

Figure S3:

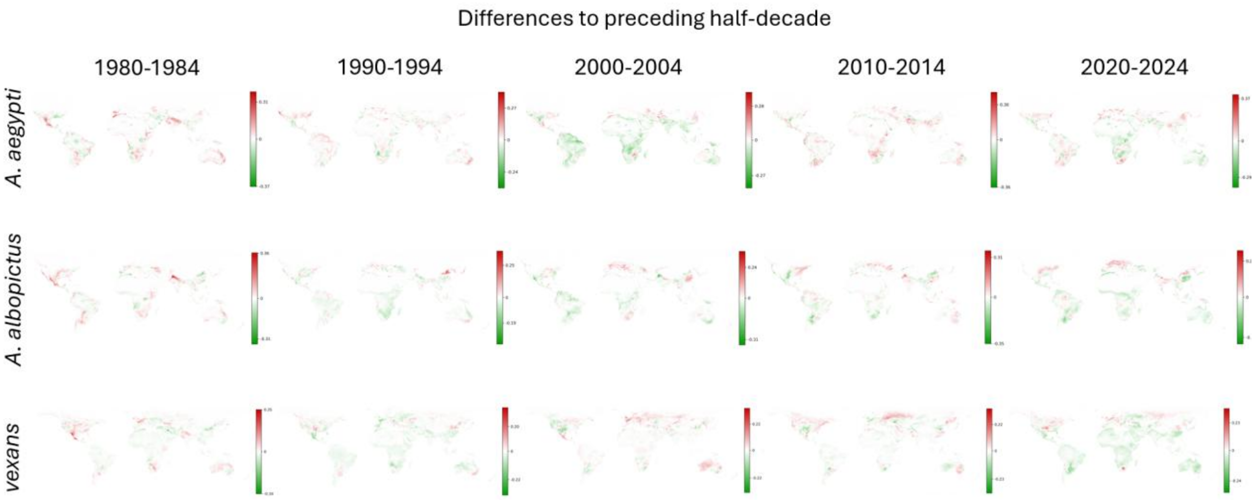

Supplementary Figure 3: Difference maps between consecutive half-decades. Differences were taken between consecutive half-decades, beginning with diff (bottom). Red indicates an increase in mosquito risk at a given location, while green indicates a decrease.

Figure S4:

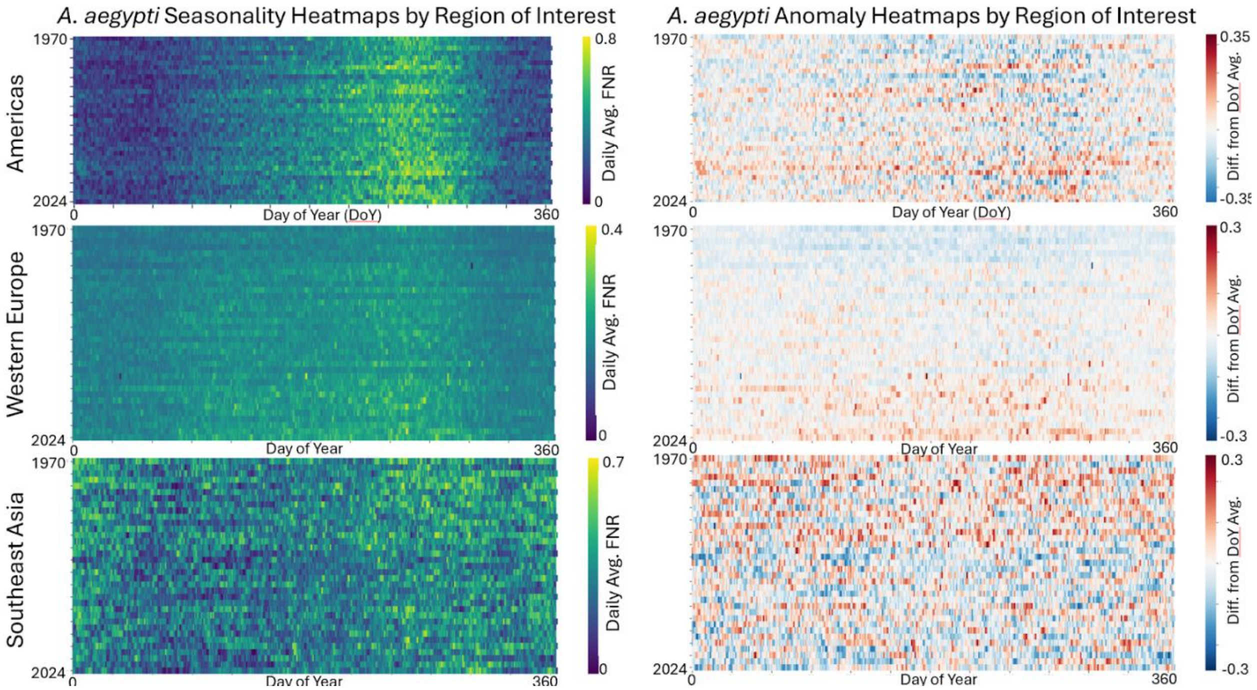

Supplementary Figure 4: Seasonality and seasonal anomaly heatmaps for *A. aegypti* by region of interest. Average FNR values are taken over the entire globe (ex-ocean) for every day of study (left). Anomaly heatmaps show the difference between a given day's average FNR value and the average across all years with the same day of year, with red indicating an increase and blue representing a decrease from the day-of-year average.

Figure S5:

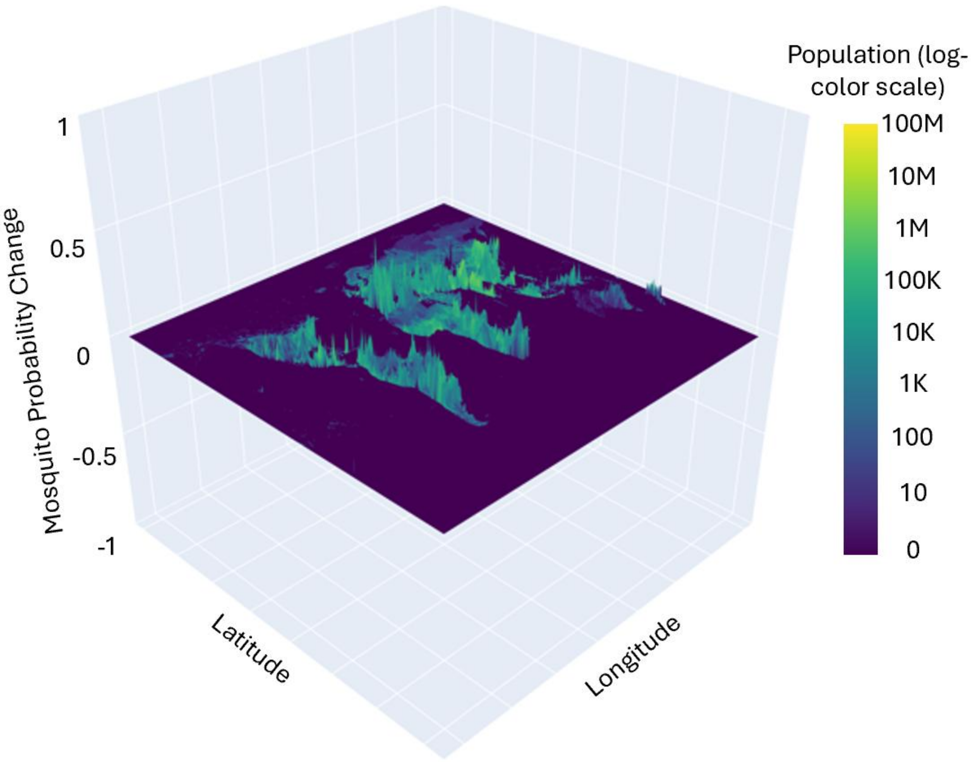

Supplementary Figure 5: 3D Plot of FNR change and corresponding population. Peaks (and troughs) represent increase (or decrease) in FNR for *A. aegypti* at a given location from 2010 to 2020. Color corresponds to the population at that location (log color scale).
