# Supplementary figures and images for: Mosquito vector ecologies are destabilizing as a result of climate change
Source: bioRxiv. 2025 Sep 17:2025.09.15.676177. Preprint. [Version 1] doi: 10.1101/2025.09.15.676177 (PMC12458471; doi:10.1101/2025.09.15.676177)

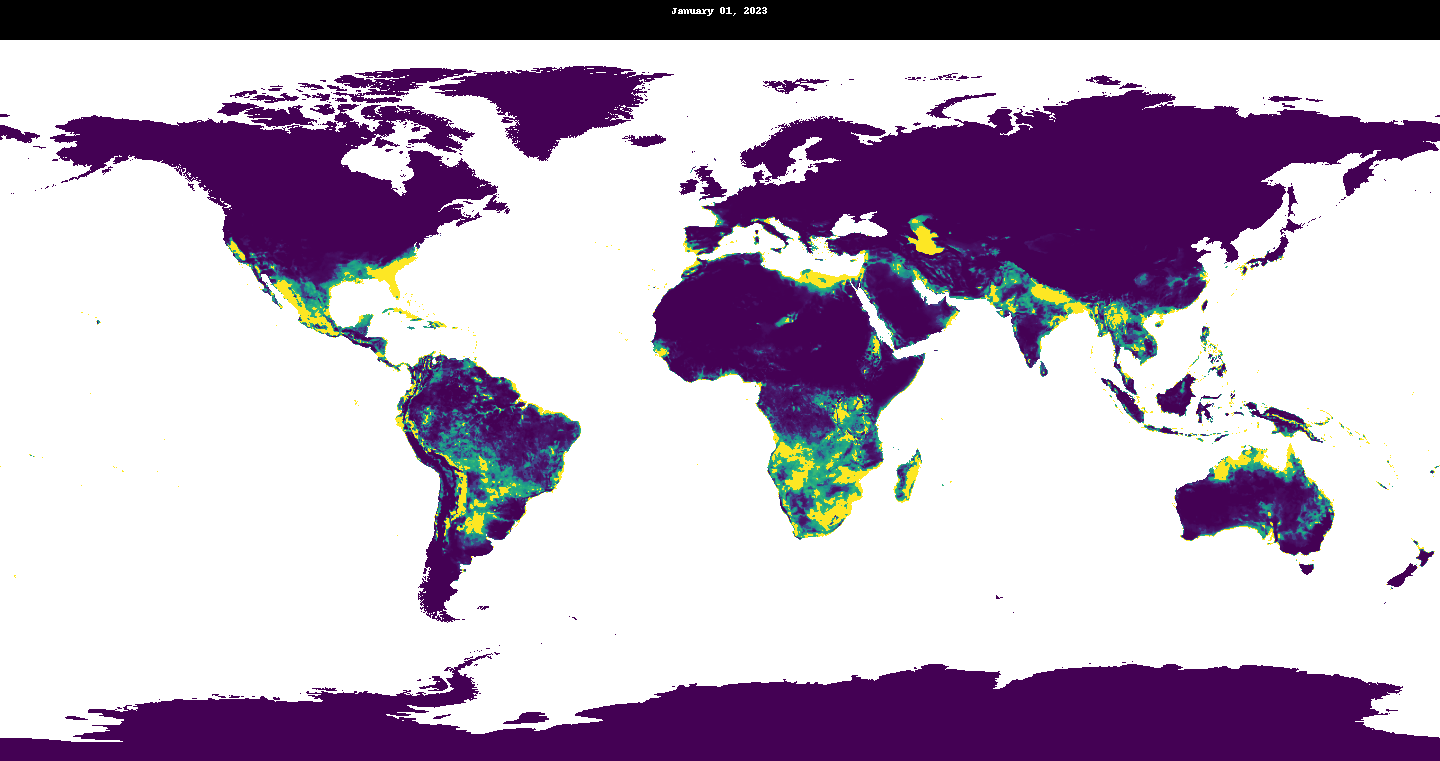

Supplement: Supplement 2 — Movies: Yearly GIFs for 2023 & 2024, all three species [file media-2.zip › Year gifs/aegypti_2023.gif]

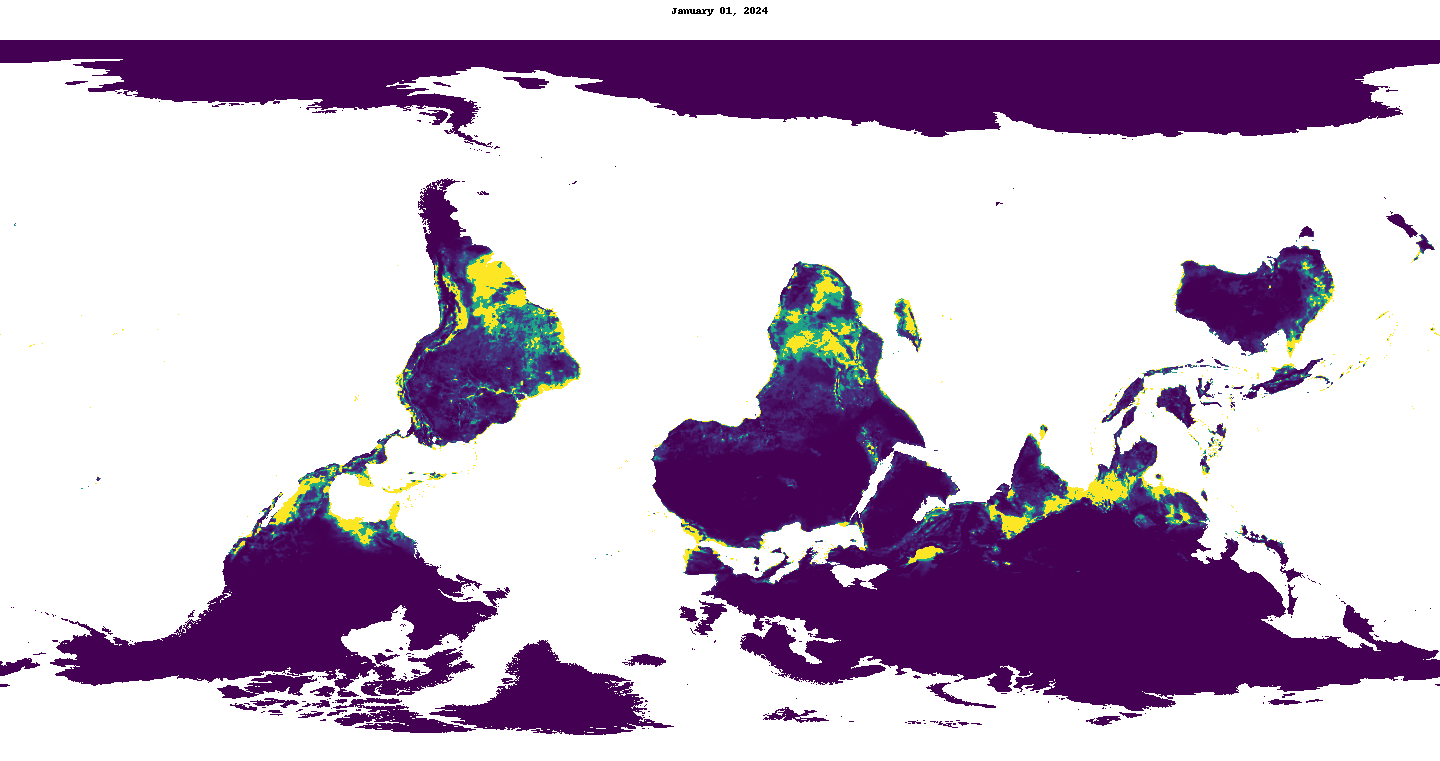

Supplement: Supplement 2 — Movies: Yearly GIFs for 2023 & 2024, all three species [file media-2.zip › Year gifs/aegypti_2024.gif]

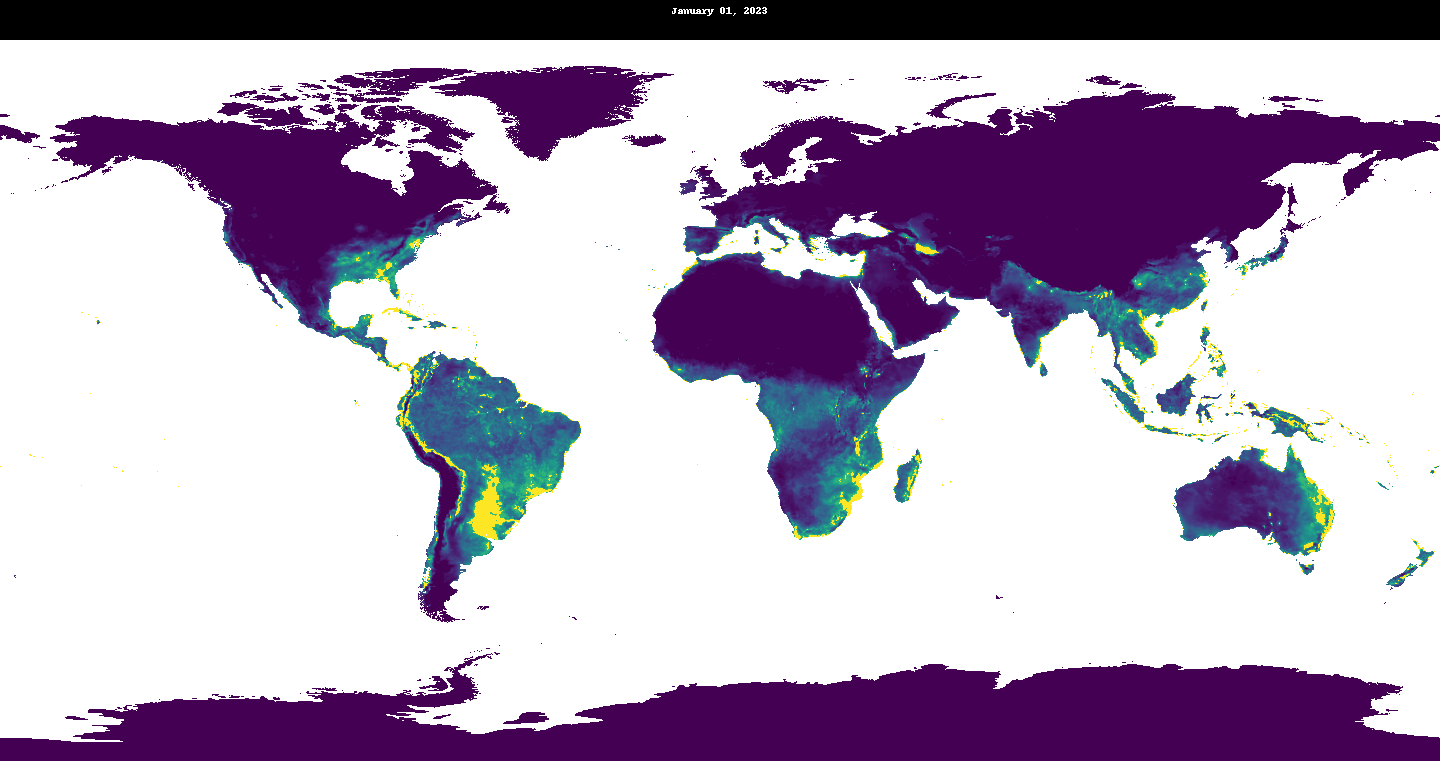

Supplement: Supplement 2 — Movies: Yearly GIFs for 2023 & 2024, all three species [file media-2.zip › Year gifs/albopictus_2023.gif]

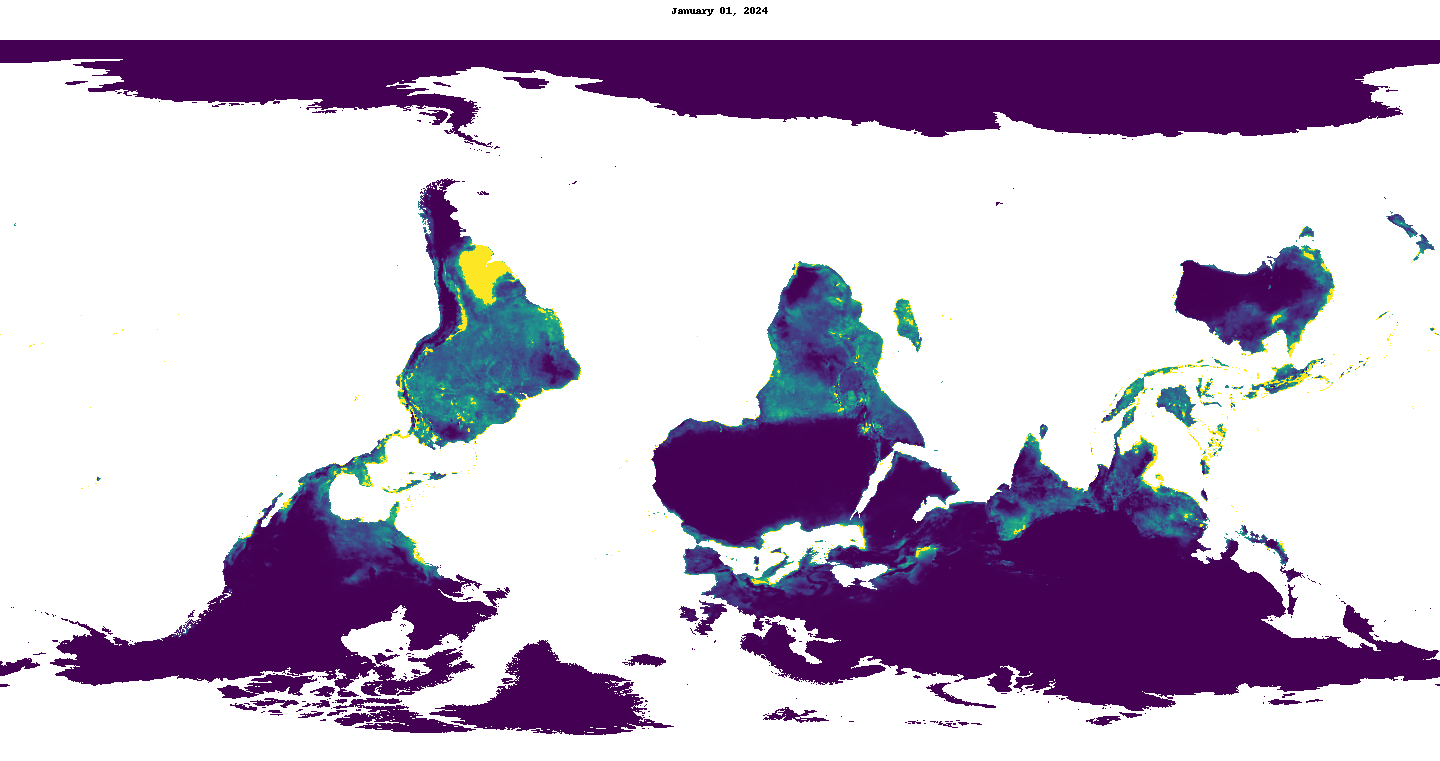

Supplement: Supplement 2 — Movies: Yearly GIFs for 2023 & 2024, all three species [file media-2.zip › Year gifs/albopictus_2024.gif]

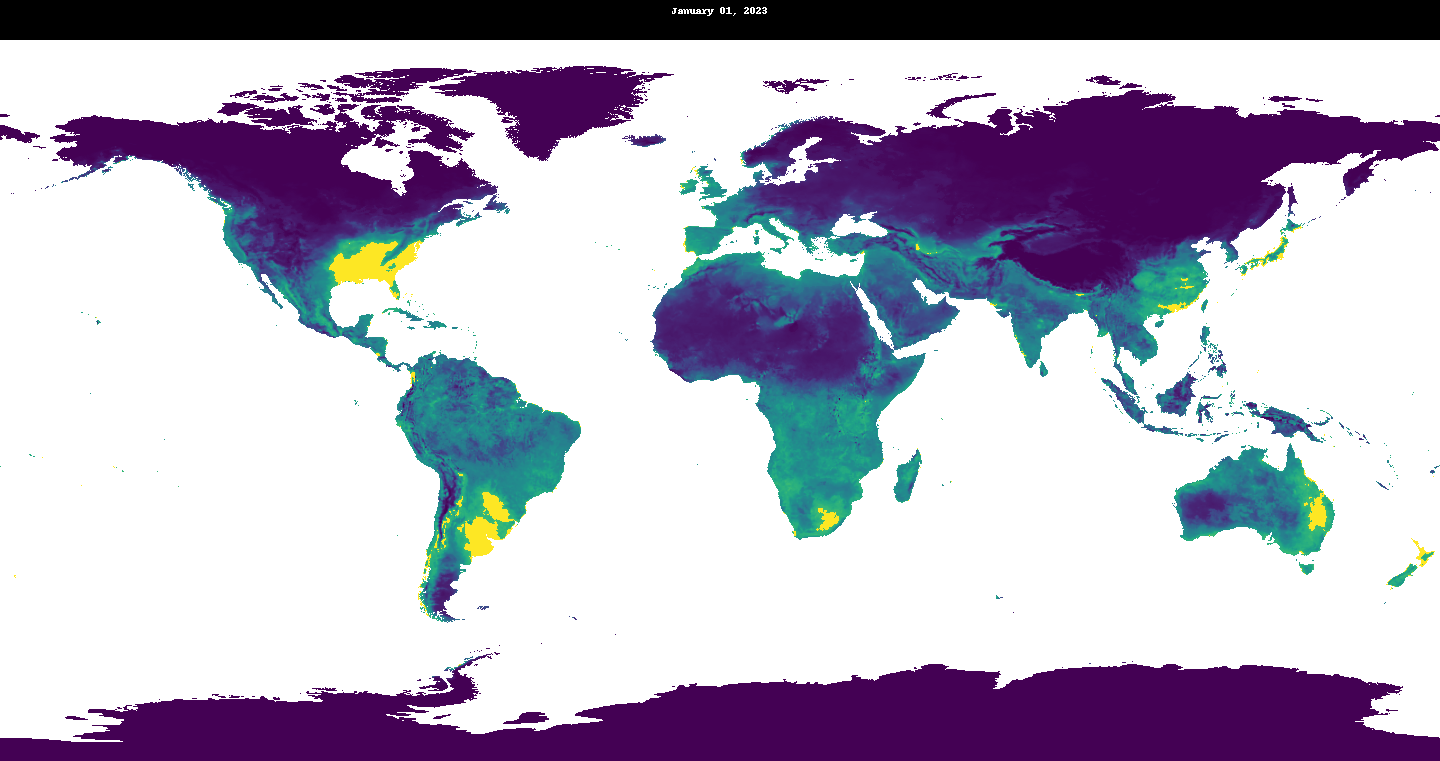

Supplement: Supplement 2 — Movies: Yearly GIFs for 2023 & 2024, all three species [file media-2.zip › Year gifs/vexans_2023.gif]

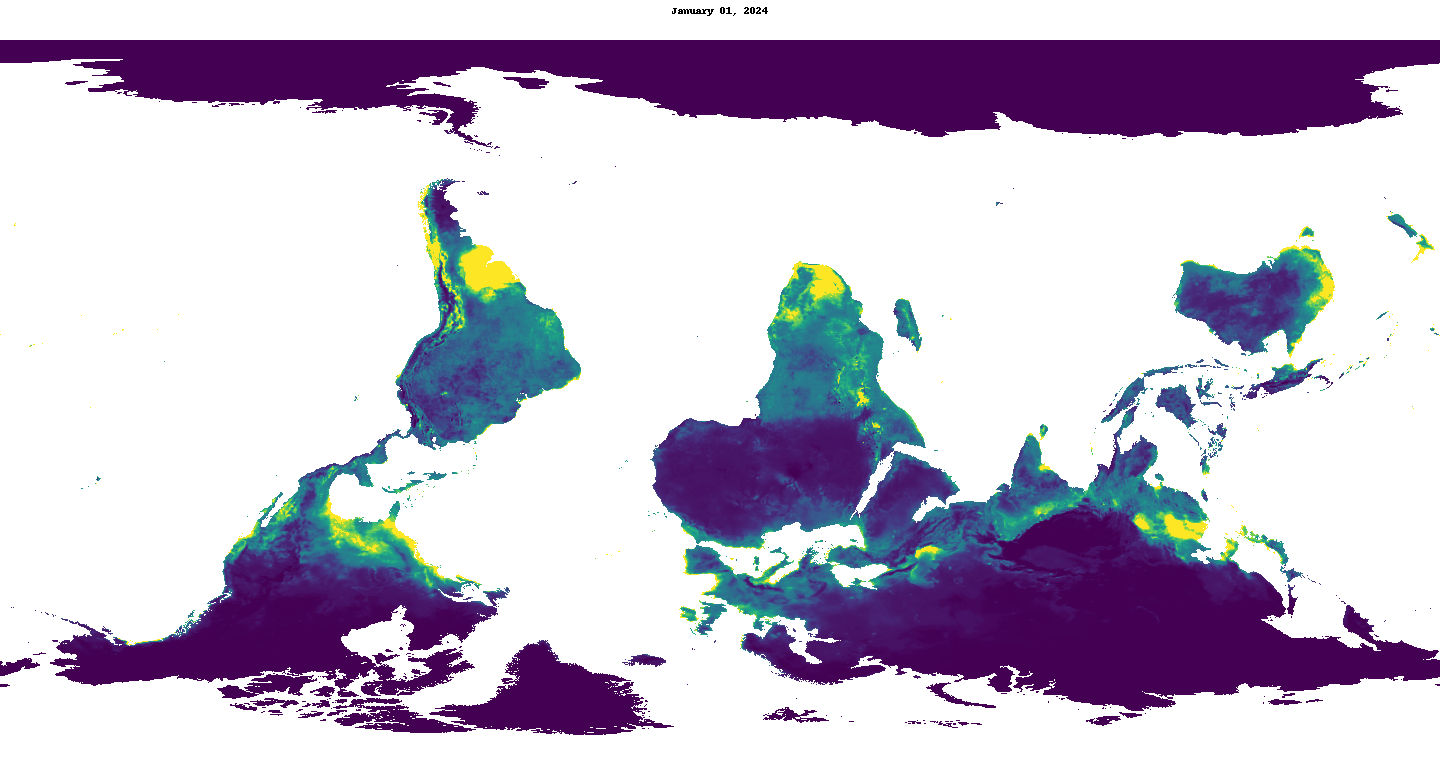

Supplement: Supplement 2 — Movies: Yearly GIFs for 2023 & 2024, all three species [file media-2.zip › Year gifs/vexans_2024.gif]
